# Magnetic Resonance Imaging Approaches for Studying Alcoholism Using Mouse Models

**Published:** 2008

**Authors:** Eilis A. Boudreau, Gang Chen, Xin Li, Christopher D. Kroenke

**Keywords:** Alcoholism, alcohol and other drug effects and consequences, animal studies, laboratory mice, mouse brain, brain function, brain structure, neuroimaging, magnetic resonance imaging (MRI), functional magnetic resonance imaging (fMRI), pharmacological magnetic resonance imaging (phMRI)

Mice are one of the most commonly used animal models of alcoholism, and extensive genetic and behavioral data related to alcohol consumption and its consequences in different strains are available. However, only recently have researchers begun to combine magnetic resonance imaging (MRI) technology with other experimental strategies to study the effects of alcohol in mice. This powerful combination enables structural and functional data of alcohol’s effects on the brain of living animals to be obtained. This article reviews the challenges associated with the use of these technologies in mice and discusses the application of these advanced technologies to mouse models of alcoholism.

## Technological Advances Allowing MRI Studies in Mice

Application of MRI approaches to mouse model studies of human diseases, including alcoholism, has been limited by the small size of the mouse brain, which results in insufficient spatial resolution. However, these technological difficulties can be overcome with the use of modern high-field (7 to 12 Tesla [T]^1^) MRI systems suitable for scanning small animals. To obtain the requisite image resolution to study the mouse brain, the size of each volume element (i.e., voxel) analyzed must be reduced by a factor of approximately 100 compared with a typical MRI examination of the human brain. This reduction in voxel size is greatly facilitated by using small magnetic field gradient coils that are capable of rapidly generating extremely steep magnetic field gradients (Mayer et al. 2007). With these small-animal gradient systems, voxel sizes of less than 1 cubic millimeter (mm^3^) can be achieved using a variety of standard MRI techniques, whereas only a subset of imaging methods (such as T1-weighted imaging) can be used to generate submillimeter voxels in a clinical MRI system.

A second technical issue affecting MRI analyses in small animals, such as mice, relates to sensitivity (or signal-to-noise ratio [SNR]). If the same MRI instrumentation were used for small-animal imaging experiments as for clinical MRI analyses, the SNR would be expected to be reduced by about 100-fold, corresponding to the 100-fold reduction in voxel volume. This potential reduction in sensitivity can be avoided by using receiver elements matched in size to the volume under study (Doty et al. 2007). Other technical modifications (i.e., increased spin polarization at high magnetic field strength) have led to further gains in sensitivity relative to clinical MRIs (de Graf et al. 2006). These factors, combined with the feasibility of performing long MRI examinations with animals, typically result in relative increases in SNR when compared with similar human experiments. As a result, small-animal MRI has seen rapid growth in neuroscience research over recent years (for reviews, see Benveniste and Blackband 2002; Van der Linden et al. 2007).

Another problem associated with performing MRI scans in rodents is that the animals typically need to be sedated, potentially causing interactions between alcohol and the anesthetic used. To address the complications of scanning anesthetized animals, King and colleagues (2005) developed procedures for scanning conscious animals without the use of anesthetic. Their original procedure involved securing the animal in a restraining device that utilized a plastic headpiece to prevent movement. However, this treatment resulted in significant stress to the animals, as indicated by increased respiratory and heart rates and increased stress hormone (i.e., corticosterone) levels. The researchers therefore developed a protocol to gradually acclimate the animal to conscious scanning conditions by repeatedly placing them in a mock-MRI setup over a period of several days. Repeated measurements confirmed that as the animals acclimated to the procedure, their respiratory and heart rates and corticosterone levels declined.

## Application of Recent Advances in Functional MRI to Mouse Models of Alcoholism

Functional MRI (fMRI) utilizes changes in the MRI signal between oxygen-rich (i.e., oxygenated) and oxygen-deprived (i.e., deoxygenated) blood to indirectly quantify alterations in blood flow associated with neuronal activity^2^ (Buxton et al. 2002). (This blood oxygen level–dependent mechanism of generating MRI contrast is termed the BOLD effect.) In a typical fMRI experiment, the MRI signal during a baseline state is compared with the MRI signal obtained following a stimulus. In an extension of this strategy, termed pharmacological MRI (phMRI), the baseline state may be compared to conditions following administration of a pharmacological agent (e.g., alcohol). With recent advances in small-animal MRI, it now is possible to study the effects of pharmacological agents, such as alcohol and other drugs (i.e., to perform phMRIs [Chen et al. 1997]), in very small animals such as mice with sufficient resolution and without the confounding factor of drug–anesthetic interactions.

**Figure 6:**
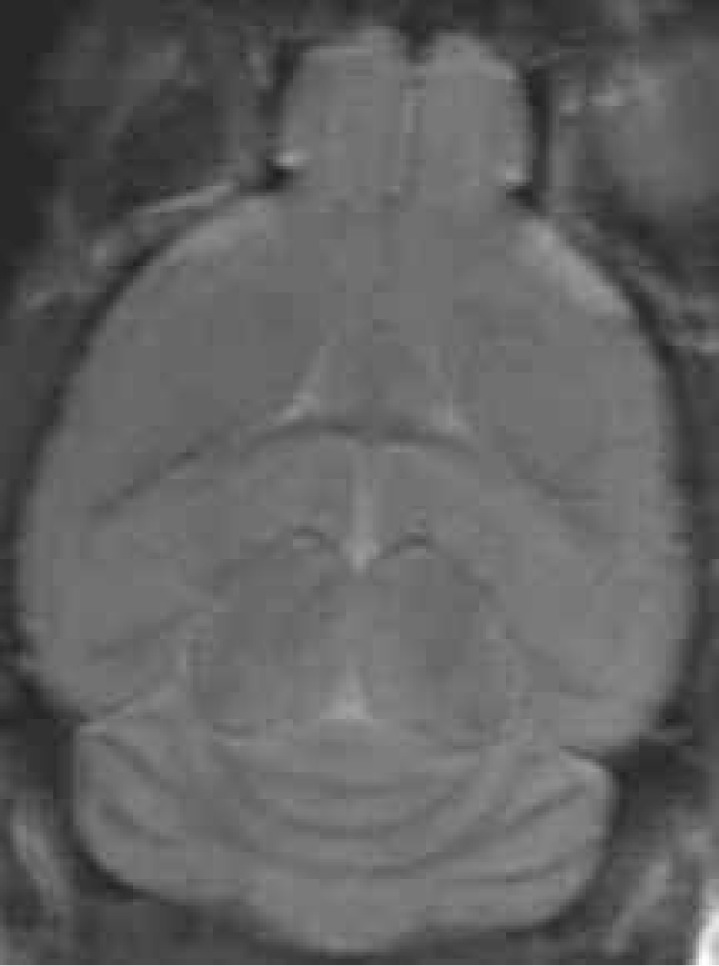
High-resolution anatomic magnetic resonance image (MRI) of the brain of a conscious mouse. This demonstrates the feasibility of conducting MRI analyses in awake mice serving as models for various aspects of human alcoholism. NOTE: The image is a T2-weighted image obtained using a 11.75 T Bruker wide-bore animal scanner. Images were acquired with in-plane resolution of 0.098 X 0.098 mm^2^, 1.0 mm slice thickness, and TR/TE = 2,773/32 ms.

Based on the acclimation strategy developed by King and colleagues (2005) for MRIs in rats, researchers have begun to modify the procedure further and develop a protocol for studying the acute response to alcohol administration in two strains of mice, called C57BL/6J and DBA/2J. These two strains were chosen because their genetic background (i.e., genotype) and behavior (i.e., phenotype) with regard to their response to alcohol have been studied extensively. For this approach, the animals also are acclimated to the scanning procedure by placing them in a mock-MRI set-up before the phMRI scanning was initiated. Initial experiments have indicated that high-quality anatomic scans of the mouse brain can be achieved at higher-field strengths (see figure 6) and that it is possible to obtain functional scans of good quality in awake, unsedated mice. Thus, this approach has great potential for future studies of alcohol’s effects on the brain using animal models.

## Conclusions

Animal models, particularly studies in mice and rats, have greatly advanced researchers’ understanding of the effects that alcohol has on the body, including the brain. Because of the animals’ small size and the associated technical challenges, however, brain imaging studies in live animals only recently have become feasible, thanks to improvements in the required instrumentation and adaptations of the experimental designs. The full potential of these technological advances is only beginning to be realized.

## Footnotes

1 The Tesla (T) is the unit of measurement for magnetic fields. For example, the strength of the Earth’s magnetic field at the equator is 31 microtesla (μT). Normal MRI systems have a magnetic field strength of 1.5 to 3 T.

2 Neurons, like other cells, require oxygen for their activity. This oxygen is delivered to the cells by the blood. The more active a brain cell or brain region is, the more oxygen it requires. Thus, brain regions of high activity are characterized by increased blood flow. Moreover, in these regions oxygen is released from the oxygenated blood into the cells, leaving the blood deoxygenated. This oxygen release can be measured because oxygenated and deoxygenated blood generates different MRI signals.
